# The Emergency Response Capacity of Plant-Based Biopharmaceutical Manufacturing-What It Is and What It Could Be

**DOI:** 10.3389/fpls.2020.594019

**Published:** 2020-10-20

**Authors:** Daniel Tusé, Somen Nandi, Karen A. McDonald, Johannes Felix Buyel

**Affiliations:** ^1^ DT/Consulting Group and GROW Biomedicine, LLC, Sacramento, CA, United States; ^2^ Department of Chemical Engineering, University of California, Davis, Davis, CA, United States; ^3^ Global HealthShare Initiative, University of California, Davis, Davis, CA, United States; ^4^ Fraunhofer Institute for Molecular Biology and Applied Ecology IME, Aachen, Germany; ^5^ Institute for Molecular Biotechnology, RWTH Aachen University, Aachen, Germany

**Keywords:** plant molecular farming, severe acute respiratory syndrome coronavirus 2, rapid scalability, regulatory approval, transient expression

## Abstract

Several epidemic and pandemic diseases have emerged over the last 20 years with increasing reach and severity. The current COVID-19 pandemic has affected most of the world’s population, causing millions of infections, hundreds of thousands of deaths, and economic disruption on a vast scale. The increasing number of casualties underlines an urgent need for the rapid delivery of therapeutics, prophylactics such as vaccines, and diagnostic reagents. Here, we review the potential of molecular farming in plants from a manufacturing perspective, focusing on the speed, capacity, safety, and potential costs of transient expression systems. We highlight current limitations in terms of the regulatory framework, as well as future opportunities to establish plant molecular farming as a global, de-centralized emergency response platform for the rapid production of biopharmaceuticals. The implications of public health emergencies on process design and costs, regulatory approval, and production speed and scale compared to conventional manufacturing platforms based on mammalian cell culture are discussed as a forward-looking strategy for future pandemic responses.

## Introduction

The impact of the COVID-19 pandemic caused by the novel severe acute respiratory syndrome coronavirus 2 (SARS-CoV-2) was foreshadowed by earlier epidemics of new or re-emerging diseases such as SARS (2002/2003), influenza (2009), Middle East Respiratory Syndrome (MERS, 2012), Ebola (2014/2015), and Zika (2016/2017) affecting localized regions (Bradley and Bryan, 2019; Kobres et al., 2019; Park et al., 2019). These events showed that novel and well-known viral diseases alike can pose a threat to global health. In 2014, an article published in *Nature Medicine* stated that the Ebola outbreak should have been “a wake-up call to the research and pharmaceutical communities, and to federal governments, of the continuing need to invest resources in the study and cure of emerging infectious diseases” (Anonymous, 2014). Recommendations and even new regulations have been implemented to reduce the risk of zoonotic viral infections (Li et al., 2019), but the extent to which these recommendations are applied and enforced on a regional and, more importantly, local level remains unclear. Furthermore, most vaccine programs for SARS, MERS, and Zika are still awaiting the fulfillment of clinical trials, sometimes more than 5 years after their initiation, due to the lack of patients (Pregelj et al., 2020). In light of this situation, and despite the call to action, the SARS-CoV-2 pandemic has resulted in nearly 20 million infections and more than 700,000 deaths at the time of writing (August 2020) based on the Johns Hopkins University Hospital global database.^1^ The economic impact of the pandemic is difficult to assess, but support programs are likely to cost more than €4 trillion (US$4.7 trillion) in the United States and EU alone. Given the immense impact at both the personal and economic levels, this review considers how the plant-based production of recombinant proteins (e.g., vaccines, therapeutics, diagnostics, and laboratory reagents) can contribute to a global response in such an emergency scenario. Several recent publications describe in broad terms how plant-made countermeasures against SARS-CoV-2 can contribute to the global COVID-19 response (Capell et al., 2020; McDonald and Holtz, 2020; Rosales-Mendoza, 2020). This review will focus primarily on process development, manufacturing considerations, and evolving regulations to identify gaps and research needs, as well as regulatory processes and/or infrastructure investments that can help to build a more resilient pandemic response system. We first highlight the technical capabilities of plants, such as the speed of transient expression, making them attractive as a first-line response to counter pandemics, and then we discuss the regulatory pathway for plant-made pharmaceuticals (PMPs) in more detail. Next, we briefly present the types of plant-derived proteins that are relevant for the prevention, treatment, or diagnosis of disease. This sets the stage for our assessment of the requirements in terms of production costs and capacity to mount a coherent response to a pandemic, given currently available infrastructure and the intellectual property (IP) landscape. We conclude by comparing plant-based expression with conventional cell culture and highlight where investments are needed to adequately respond to pandemic diseases in the future. Due to the quickly evolving information about the pandemic, our statements are supported in some instances by data obtained from web sites (e.g., governmental publications). Accordingly, the scientific reliability has to be treated with caution in these cases.

## Technical Aspects of Plant-Based Production Systems

### Screening of Product Candidates

The development of a protein-based vaccine, therapeutic, or diagnostic reagent for a novel disease requires the screening of numerous expression cassettes, for example, to identify suitable regulatory elements (e.g., promoters) that achieve high levels of product accumulation, a sub-cellular compartment that ensures product integrity, as well as different product candidates to identify the most active and most amenable to manufacturing in plants (Buyel et al., 2013a; Kohli et al., 2015; DiCara et al., 2018; Spiegel et al., 2019; Kerwin et al., 2020). A major advantage of plants in this respect is the ability to test multiple product candidates and expression cassettes in parallel by the simple injection or infiltration of leaves or leaf sections with a panel of *Agrobacterium tumefaciens* clones carrying each variant cassette as part of the transferred DNA (T-DNA) in a binary transformation vector (Piotrzkowski et al., 2012; Norkunas et al., 2018; Rademacher et al., 2019). This procedure does not require sterile conditions, transfection reagents, or skilled staff, and can, therefore, be conducted in standard biosafety level 1 (BSL 1) laboratories all over the world. The method can produce samples of even complex proteins such as glycosylated monoclonal antibodies (mAbs) for analysis ~14 days after the protein sequence is available. With product accumulation in the range of 0.1–4.0 g kg^−1^ biomass (Sainsbury and Lomonossoff, 2008; Zischewski et al., 2015; Yamamoto et al., 2018), larger-scale quantities (several grams) can be supplied after 4–8 weeks (Shoji et al., 2012), making this approach ideal for emergency responses to sudden disease outbreaks. Potential bottlenecks include the preparation of sufficiently large candidate libraries, ideally in an automated manner as described for conventional expression systems, and the infiltration of plants with a large number (>100) of candidates. Also, leaf-based expression can result in a coefficient of variation (CV) >20% in terms of recombinant protein accumulation, which reduces the reliability of expression data (Buyel and Fischer, 2014a). The variability issue has been addressed to some extent by a parallelized leaf-disc assay at the cost of a further reduction in sample throughput (Piotrzkowski et al., 2012).

The reproducibility of screening was improved in 2018 by the development of plant cell pack technology, in which plant cell suspension cultures deprived of medium are used to form a plant tissue surrogate that can be infiltrated with *A. tumefaciens* in a 96-well microtiter plate format to produce milligram quantities of protein in an automated, high-throughput manner. The costs (without analysis) can be as low as €0.50 (US$0.60) per 60-mg sample with a product accumulation of ~100 mg kg^−1^ and can typically result in a CV of <5% (Gengenbach et al., 2020). These costs include the fermenter-based upstream production of plant cells as well as all materials and labor. The system can be integrated with the cloning of large candidate libraries, allowing a throughput of >1,000 samples per week, and protein is produced 3 days after infiltration. The translatability of cell pack data to intact plants was successfully demonstrated for three mAbs and several other proteins, including a toxin (Gengenbach et al., 2019; Rademacher et al., 2019). Therefore, cell packs allow the rapid and automated screening of product candidates such as vaccines and diagnostic reagents. In addition to recombinant proteins, the technology can, in principle, also be used to produce virus-like particles (VLPs) based on plant viruses, which further broadens its applicability for screening and product evaluation but, to our knowledge, according results had not been published as of September 2020. In the future, plant cell packs could be combined with a recently developed method for rapid gene transfer to plant cells using carbon nanotubes (Demirer et al., 2019). Such a combination would not be dependent on bacteria for cloning (*Escherichia coli*) or gene transfer to plant cells (*A. tumefaciens*), thereby reducing the overall duration of the process by an additional 2–3 days (Demirer et al., 2019).

For the rapid screening of even larger numbers of candidates, cost-efficient cell-free lysates based on plant cells have been developed and are commercially available in a ready-to-use kit format. Proteins can be synthesized in ~24 h, potentially in 384-well plates, and the yields expressed as recombinant protein mass per volume of cell lysate can reach 3 mg ml^−1^ (Buntru et al., 2015). Given costs of ~€1,160 (US$1,363) ml^−1^ according to the manufacturer LenioBio (Germany), this translates to ~€400 ($470) mg^−1^ protein, an order of magnitude less expensive than the SP6 system (Promega, United States), which achieves 0.1 mg ml^−1^ at a cost of ~€360 ($423) ml^−1^ (€3,600 or $4,230 mg^−1^) based on the company’s claims. Protocol duration and necessary labor are comparable between the two systems and so are the proteins used to demonstrate high expression, e.g., luciferase. However, the scalability of the plant-cell lysates is currently limited to several hundred milliliters, and transferability to intact plants has yet to be demonstrated, i.e., information about how well product accumulation in lysates correlates with that in plant tissues. Such correlations can then form the basis to scale-up lysate-based production to good manufacturing practice (GMP)-compliant manufacturing in plants using existing facilities. Therefore, the cell packs are currently the most appealing screening system due to their favorable balance of speed, throughput, and translatability to whole plants for large-scale production.

In any pandemic, the pathogen genome has to be sequenced, made publically available, and freely disseminated in the global scientific community (for which there are currently no well-defined workflows) to accelerate therapeutic and vaccine development. Once sequence information is available, a high priority is the rapid development, synthesis, and distribution of DNA sequences coding for individual viral open reading frames. These reagents are not only important for screening subunit vaccine targets but also as enabling tools for research into the structure, function, stability, and detection of the virus (Khailany et al., 2020). Because many viral pathogens (including SARS-CoV-2) mutate over time, the sequencing of clinical virus samples is equally important to enable the development of countermeasures to keep pace with virus evolution (Kupferschmidt, 2020). To ensure the broadest impact, the gene constructs must be codon optimized for expression in a variety of hosts (Hanson and Coller, 2018); cloned into plasmids with appropriate promoters, purification tags, and watermark sequences to identify them as synthetic and so that their origin can be verified (Liss et al., 2012); and made widely available at minimal cost to researchers around the world. Not-for-profit plasmid repositories, such as Addgene and DNASU, in cooperation with global academic and industry contributors, play an important role in providing and sharing these reagents. However, the availability of codon-optimized genes for plants and the corresponding expression systems is often limited (Webster et al., 2017). For example, there were 41,247 mammalian, 16,560 bacterial, and 4,721 yeast expression vectors in the Addgene collection as of August 2020, but only 1,821 for plants, none of which contained SARS-CoV-2 proteins. Sharing plant-optimized SARS-CoV-2 synthetic biology resources among the academic and industry research community working on PMPs would further accelerate the response to this pandemic disease.

Screening and process development can also be expedited by using modeling tools to identify relevant parameter combinations for experimental testing. For example, initial attempts have been made to establish correlations between genetic elements or protein structures and product accumulation in plants (Buyel et al., 2013a; Jansing and Buyel, 2019). Similarly, heuristic and model-based predictions can be used to optimize downstream processing (DSP) unit operations including chromatography (Buyel et al., 2013b; Buyel and Fischer, 2014c; Alam et al., 2018). Because protein accumulation often depends on multiple parameters, it is typically more challenging to model than chromatography and probably needs to rely on data-driven rather than mechanistic models. Based on results obtained for antibody production, a combination of descriptive and mechanistic models can reduce the number of experiments and thus the development time by 75% (Möller et al., 2019), which is a substantial gain when trying to counteract a global pandemic such as COVID-19. These models are particularly useful if combined with the high-throughput experiments described above. Techno-economic assessment (TEA) computer-aided design tools, based on engineering process models, can be used to design and size process equipment, solve material and energy balances, generate process flowsheets, establish scheduling, and identify process bottlenecks. TEA models have been developed and are publicly available for a variety of plant-based biomanufacturing facilities, including whole plant and plant cell bioreactor processes for production of mAbs (Nandi et al., 2016), antiviral lectins (Alam et al., 2018), therapeutics (Tusé et al., 2014; Corbin et al., 2020), and antimicrobial peptides (McNulty et al., 2020). These tools are particularly useful for the development of new processes because they can indicate which areas would benefit most from focused research and development (R&D) efforts to increase throughput, reduce process mass intensity, and minimize overall production costs.

### Transient Protein Expression in Plants

The rapid production of protein-based countermeasures for SARS-CoV-2 will most likely, at least initially, require biomanufacturing processes based on transient expression rather than stable transgenic lines. Options include the transient transfection of mammalian cells (Gutiérrez-Granados et al., 2018), baculovirus-infected insect cell expression systems (Contreras-Gomez et al., 2014), cell-free expression systems for *in vitro* transcription and translation (Zemella et al., 2015), and transient expression in plants (Sainsbury, 2020). The longer-term production of these countermeasures may rely on mammalian or plant cell lines and/or transgenic plants, in which the expression cassette has been stably integrated into the host genome, but these will take months or even years to develop, optimize, and scale-up. Among the available transient expression systems, only plants can be scaled-up to meet the demand for COVID-19 countermeasures without the need for extensive supply chains and/or complex and expensive infrastructure, thus ensuring low production costs (Nandi et al., 2016). These manufacturing processes typically use *Nicotiana benthamiana* (a relative of tobacco) as the production host and each plant can be regarded as a biodegradable, single-use bioreactor (Buyel, 2018). The plants are grown either in greenhouses or indoors, either hydroponically or in a growth substrate, often in multiple layers to minimize the facility footprint, and under artificial lighting such as LEDs. In North America, large-scale commercial PMP facilities have been built in Bryan, TX (Caliber Biotherapeutics, acquired by iBio), Owensboro, KY (Kentucky Bioprocessing), Durham, NC (Medicago), and Quebec, Canada (Medicago; Pogue et al., 2010; Holtz et al., 2015; Lomonossoff and D’Aoust, 2016). The plants are grown from seed until they reach 4–6 weeks of age before transient expression, which is typically achieved by infiltration using recombinant *A. tumefaciens* carrying the expression cassette (as described above for screening) or by the introduction of a viral expression vector such as tobacco mosaic virus (TMV), for example, the GENEWARE platform (Pogue et al., 2010). For transient expression by infiltration with *A. tumefaciens*, the plants are turned upside down and the aerial portions are submerged in the bacterial suspension. A moderate vacuum is applied for a few minutes, and when it is released, the bacteria are drawn into the interstitial spaces within the leaves. The plants are removed from the suspension and moved to an incubation room/chamber for 5–7 days for recombinant protein production. A recent adaptation of this process replaces vacuum infiltration with the aerial application of the *A. tumefaciens* suspension mixed with a surfactant. The reduced surface tension of the carrier solution allows the bacteria to enter the stomata, achieving a similar effect to agroinfiltration (Hahn et al., 2015). This agrospray strategy can be applied anywhere, thus removing the need for vacuum infiltrators and associated equipment (and transfer to and from the infiltration units). For transient expression using viral vectors, the viral suspension is mixed with an abrasive for application to the leaves using a pressurized spray, and the plants are incubated for 6–12 days as the recombinant protein is produced. Large-scale production facilities have an inventory of plants at various stages of growth and they are processed (by infiltration or inoculation) in batches. Depending on the batch size (the number of plants per batch), the vacuum infiltration throughput, and the target protein production kinetics, the infiltration/incubation process time is 5–8 days. The inoculation/incubation process is slightly longer at 6–13 days.

The overall batch time from seeding to harvest is 33–55 days depending on the optimal plant age, transient expression method, and target protein production kinetics (Pogue et al., 2010; Holtz et al., 2015; Lomonossoff and D’Aoust, 2016). Importantly, plant growth can be de-coupled from infiltration, so that the plants are kept at the ready for instant use, which reduces the effective first-reaction batch time from gene to product to ~10–15 days if a platform downstream process is available (e.g., Protein A purification for mAbs). The time between batches can be reduced even further to match the longest unit operation in the upstream or downstream process. The number of plants available under normal operational scenarios is limited to avoid expenditure, but more plants can be seeded and made available in the event of a pandemic emergency. This would allow various urgent manufacturing scenarios to be realized, for example, the provision of a vaccine candidate or other prophylactic to first-line response staff.

### Processing of Plant Biomass

The speed of transient expression in plants allows the rapid adaptation of a product even when the process has already reached manufacturing scale. For example, decisions about the nature of the recombinant protein product can be made as little as 2 weeks before harvest because the cultivation of bacteria (including a seed train) takes less than 7 days (Houdelet et al., 2017) and the post-infiltration incubation of plants takes ~5–7 days. By using large-scale cryo-stocks of ready-to-use *A. tumefaciens*, the decision can be delayed until the day of infiltration and thus 5–7 days before harvesting the biomass (Spiegel et al., 2019). This flexibility is desirable in an early pandemic scenario because the latest information on improved drug properties can be channeled directly into production, for example, to produce gram quantities of protein that are required for safety assessment, pre-clinical and clinical testing, or even compassionate use if the fatality rate of a disease is high (see section “European guidance for COVID-19 medicine developers and companies” below).

Although infiltration is typically a discontinuous process requiring stainless-steel equipment due to the vacuum that must be applied to plants submerged in the bacterial suspension, most other steps in the production of PMPs can be designed for continuous operation, incorporating single-use equipment and thus complying with the proposed concept for biofacilities of the future (Klutz et al., 2015). Accordingly, continuous harvesting and extraction can be carried out using appropriate equipment such as screw presses (Buyel and Fischer, 2015), whereas continuous filtration and chromatography can take advantage of the same equipment successfully used with microbial and mammalian cell cultures (David et al., 2020). Therefore, plant-based production platforms can benefit from the same >4-fold increase in space-time yield (e.g., measured in g L^−1^ d^−1^ or g m^−2^ d^−1^) that can be achieved by continuous processing with conventional cell-based systems (Arnold et al., 2019). As a consequence, a larger amount of product can be delivered earlier, which can help to prevent the disease from spreading once a vaccine becomes available.

In addition to conventional chromatography, several generic purification strategies have been developed to rapidly isolate products from crude plant extracts in a cost-effective manner (Rosenberg et al., 2015; Buyel et al., 2016). Due to their generic nature, these strategies typically require little optimization and can immediately be applied to products meeting the necessary requirements, which reduces the time needed to respond to a new disease. For example, purification by ultrafiltration/diafiltration is attractive for both small (<30 kDa) and large (>500 kDa) molecules because they can be separated from plant host cell proteins (HCPs), which are typically 100–450 kDa in size, under gentle conditions such as neutral pH to ensure efficient recovery (Opdensteinen et al., 2018). This technique can also be used for simultaneous volume reduction and optional buffer exchange, reducing the overall process time and ensuring compatibility with subsequent chromatography steps. HCP removal triggered by increasing the temperature (~65°C) and/or reducing the pH (pH < 4.5) is mostly limited to stable proteins such as antibodies, and especially, the former method may require extended product characterization to ensure the function of products, such as vaccine candidates, is not compromised (Beiss et al., 2015; Menzel et al., 2018). The fusion of purification tags to a protein product can be tempting to accelerate process development when time is pressing during an ongoing pandemic. These tags can stabilize target proteins *in planta* while also facilitating purification by affinity chromatography or non-chromatographic methods such as aqueous two-phase systems (Bornhorst and Falke, 2010; Reuter et al., 2014). On the downside, such tags may trigger unwanted aggregation or immune responses that can reduce product activity or even safety (Khan et al., 2012). Some tags may be approved in certain circumstances (Jin et al., 2017), but their immunogenicity may depend on the context of the fusion protein.

The substantial toolkit available for rapid plant biomass processing and the adaptation of even large-scale plant-based production processes to new protein products ensure that plants can be used to respond to pandemic diseases with at least an equivalent development time and, in most cases, a much shorter one than conventional cell-based platforms. Although genetic vaccines for SARS-CoV-2 have been produced quickly (e.g., mRNA vaccines by Pfizer/BioNTech and Moderna/NIAID), they have never been manufactured at the scale needed to address a pandemic and their stability during transport and deployment to developing world regions remains to be shown.

## Regulatory Considerations for Product Approval and Deployment During Public Health Emergencies

### Regulatory Oversight During Non-emergency Situations

Regulatory oversight is a major and time-consuming component of any drug development program, and regulatory agencies have needed to revise internal and external procedures in order to adapt normal schedules for the rapid decision-making necessary during emergency situations. Just as important as rapid methods to express, prototype, optimize, produce, and scale new products are the streamlining of regulatory procedures to maximize the technical advantages offered by the speed and flexibility of plants and other high-performance manufacturing systems. Guidelines issued by regulatory agencies for the development of new products, or the repurposing of existing products for new indications, include criteria for product manufacturing and characterization, containment and mitigation of environmental risks, stage-wise safety determination, clinical demonstration of safety and efficacy, and various mechanisms for product licensure or approval to deploy the products and achieve the desired public health benefit.

Regardless of which manufacturing platform is employed, the complexity of product development requires that continuous scrutiny is applied from preclinical research to drug approval and post-market surveillance, thus ensuring that the public does not incur an undue safety risk and that products ultimately reaching the market consistently conform to their label claims. These goals are common to regulatory agencies worldwide, and higher convergence exists in regions that have adopted the harmonization of standards (e.g., the United States, EU, and Japan) as defined by the International Council for Harmonization (ICH),^2^ in key product areas including quality, safety, and efficacy.

#### Summary of the United States and European Regulatory Approval Processes

Both the United States and the EU have stringent pharmaceutical product quality and clinical development requirements, as well as regulatory mechanisms to ensure product quality and public safety. Differences and similarities between regional systems have been discussed elsewhere (Downing et al., 2012; Sparrow et al., 2013; van Norman, 2016; Chiodin et al., 2019; Detela and Lodge, 2019) and are only summarized here.

Stated simply, the United States, EU, and other jurisdictions follow generally a two-stage regulatory process, comprising (a) clinical research authorization and monitoring and (b) result’s review and marketing approval. The first stage involves the initiation of clinical research *via* submission of an Investigational New Drug (IND) application in the United States or its analogous Clinical Trial Application (CTA) in Europe. At the preclinical-clinical translational interphase of product development, a sponsor (applicant) must formally inform a regulatory agency of its intention to develop a new product and the methods and endpoints it will use to assess clinical safety and preliminary pharmacologic activity (e.g., a Phase I clinical study). Because the EU is a collective of independent Member States, the CTA can be submitted to a country-specific (national) regulatory agency that will oversee development of the new product.

The regulatory systems of the EU and the United States both allow pre-submission consultation on the proposed development programs *via* discussions with regulatory agencies or expert national bodies. These are known as pre-IND (PIND) meetings in the United States (FDA, 2017, 2020) and Investigational Medicinal Product Dossier (IMPD)^3^ discussions in the EU. These meetings serve to guide the structure of the clinical programs and can substantially reduce the risk of regulatory delays as the programs begin. PIND meetings are common albeit not required, whereas IMPD discussions are often necessary prior to CTA submission. At intermediate stages of clinical development (e.g., Phase II dose and schedule optimization studies), pauses for regulatory review must be added between clinical study phases. Such End of Phase (EOP) review times may range from one to several months depending on the technology and disease indication. In advanced stages of product development after pivotal, placebo-controlled randomized Phase III studies are complete, drug approval requests that typically require extensive time (see below) for review and decision-making on the part of the regulatory agencies.

In the United States, the Food and Drug Administration (FDA) controls the centralized marketing approval/authorization/licensing (depending on product class and indication) of a new product, a process that requires in-depth review and acceptance of a New Drug Application (NDA) for chemical entities, or a Biologics License Application (BLA) for biologics, the latter including PMP proteins. The EU follows both decentralized (national) processes as well as centralized procedures covering all Member States. The Committee for Medicinal Products for Human Use (CHMP), part of the European Medicines Agency (EMA), has responsibilities similar to those of the FDA and plays a key role in the provision of scientific advice, evaluation of medicines at the national level for conformance with harmonized positions across the EU, and the centralized approval of new products for market entry in all Member States.

#### Regulatory Approval Is a Slow and Meticulous Process by Design

The statute-conformance review procedures practiced by the regulatory agencies require considerable time because the laws were established to focus on patient safety, product quality, verification of efficacy, and truth in labeling. The median times required by the FDA, EMA, and Health Canada for full review of NDA applications were reported to be 322, 366, and 352 days, respectively (Downing et al., 2012; van Norman, 2016). Collectively, typical interactions with regulatory agencies will add more than 1 year to a drug development program. Although these regulatory timelines are the *status quo* during normal times, they are clearly incongruous with the needs for rapid review, approval, and deployment of new products in emergency use scenarios, such as emerging pandemics.

### Regulation of PMP Products in the United States and Europe

Plant-made intermediates, including reagents for diagnostics, antigens for vaccines, and bioactive proteins for prophylactic and therapeutic medical interventions, as well as the final products containing them, are subject to the same regulatory oversight and marketing approval pathways as other pharmaceutical products. However, the manufacturing environment as well as the peculiarities of the plant-made active pharmaceutical ingredient (API) can affect the nature and extent of requirements for compliance with various statutes, which in turn will influence the speed of development and approval. In general, the more contained the manufacturing process and the higher the quality and safety of the API, the easier it has been to move products along the development pipeline. Guidance documents on quality requirements for plant-made biomedical products exist and have provided a framework for development and marketing approval (FDA, 2002; EMA, 2006).

Upstream processes that use whole plants grown indoors under controlled conditions, including plant cell culture methods, followed by controlled and contained downstream purification, have fared best under regulatory scrutiny. This is especially true for processes that use non-food plants such as *Nicotiana* species as expression hosts. The backlash over the Prodigene incident of 2002 in the United States has refocused subsequent development efforts on contained environments (Ellstrand, 2003). In the United States, field-based production is possible and even practiced, but such processes require additional permits and scrutiny by the United States Department of Agriculture (USDA). In May 2020, to encourage innovation and reduce the regulatory burden on the industry, the USDA’s Agricultural Plant Health Inspection Service (APHIS) revised legislation covering the interstate movement or release of genetically modified organisms (GMOs) into the environment in an effort to regulate such practices with higher precision [SECURE Rule revision of 7 Code of Federal Regulations (CFR) 340].^4^ The revision will be implemented in steps (final implementation is scheduled for October 2021) and could facilitate the field-based production of PMPs.

In contrast, the production of PMPs using GMOs or transient expression in the field comes under heavy regulatory scrutiny in the EU, and several statutes have been developed to minimize environmental, food, and public risk. Many of these regulations focus on the use of food species as hosts. The major perceived risks of open-field cultivation are the contamination of the food/feed chain, and gene transfer between GM and non-GM plants. This is true today even though containment and mitigation technologies have evolved substantially since those statutes were first conceived, with the advent and implementation of transient and selective expression methods; new plant breeding technologies; use of non-food species; and physical, spatial, and temporal confinement (Passmore, 2012; Sparrow et al., 2013; Menary et al., 2020).

The United States and the EU differ in their philosophy and practice for the regulation of PMP products. In the United States, regulatory scrutiny is at the product level, with less focus on how the product is manufactured. In the EU, much more focus is placed on assessing how well a manufacturing process conforms to existing statutes. Therefore, in the United States, PMP products and reagents are regulated under pre-existing sections of the United States CFR, principally under various parts of Title 21 (Food and Drugs), which also apply to conventionally sourced products. These include current good manufacturing practice (cGMP) covered by 21 CFR Parts 210 and 211, good laboratory practice (GLP) toxicology (21 CFR 58), and a collection of good clinical practice (CGP) requirements specified by the ICH and accepted by the FDA (especially ICH E6 R1, R2 and draft R3). In the United States, upstream plant cultivation in containment can be practiced using qualified methods to ensure consistency of vector, raw materials, and cultivation procedures and/or, depending on the product, under good agricultural and collection practices (GACP). For PMP products, cGMP requirements do not come into play until the biomass is disrupted in a fluid vehicle to create a process stream. All process operations from that point forward, from crude hydrolysate to bulk drug substance and final drug product, are guided by 21 CFR 210/211 (cGMP).

In Europe, biopharmaceuticals regardless of manufacturing platform are regulated by the EMA, and the Medicines and Healthcare products Regulatory Agency (MHRA) in the United Kingdom. Pharmaceuticals from GM plants must adhere to the same regulations as all other biotechnology-derived drugs. These guidelines are largely specified by the European Commission (EC) in Directive 2001/83/EC and Regulation (EC) No 726/2004. However, upstream production in plants must also comply with additional statutes. Cultivation of GM plants in the field constitutes an environmental release and has been regulated by the EC under Directive 2001/18/EC and 1829/2003/EC if the crop can be used as food/feed (Passmore, 2012). The production of PMPs using whole plants in greenhouses or cell cultures in bioreactors is regulated by the “Contained Use” Directive 2009/41/EC, which are far less stringent than an environmental release and do not necessitate a fully-fledged environmental risk assessment. Essentially, the manufacturing site is licensed for contained use and production proceeds in a similar manner as a conventional facility using microbial or mammalian cells as the production platform.

With respect to GMP compliance, the major differentiator between the regulation of PMP products and the same or similar products manufactured using other platforms is the upstream production process. This is because many of the DSP techniques are product-dependent and, therefore, similar regardless of the platform, including most of the DSP equipment, with which regulatory agencies are already familiar. Of course, the APIs themselves must be fully characterized and shown to meet designated criteria in their specification, but this applies to all products regardless of source.

### Regulatory Oversight During Public Health Emergency Situations

During a health emergency, such as the COVID-19 pandemic, regulatory agencies worldwide have re-assessed guidelines and restructured their requirements to enable the accelerated review of clinical study proposals, to facilitate clinical studies of safety and efficacy, and to expedite the manufacturing and deployment of re-purposed approved drugs as well as novel products (Tables 1 and 2). These revised regulatory procedures could be implemented again in future emergency situations. It is also possible that some of the streamlined procedures that can expedite product development and regulatory review and approval will remain in place even in the absence of a health emergency, permanently eliminating certain redundancies and bureaucratic requirements. Changes in the United States and European regulatory processes are highlighted, with a cautionary note that these modified procedures are subject to constant review and revision to reflect an evolving public health situation.

**Table 1:** United States Food and Drug Administration (FDA) Coronavirus Treatment Acceleration Program (CTAP) emergency response timelines.

Task or function	Response time to sponsor’s request	
	Typical	Emergency
Providing information on regulatory processes to develop or evaluate new drug and biologic therapies	<30 days	1 day
Providing rapid, interactive input on most development plans (e.g., PIND summary documents)	<60 days	<72 h
Providing ultra-rapid review and comments on proposed clinical protocols	Variable (case specific)	<24 h (case specific)
Completing review of single-patient expanded access requests	Variable (case specific)	<3 h
Working closely with applicants and other regulatory agencies to expedite quality assessments for products to treat COVID-19 patients and to transfer manufacturing to alternative or new sites to avoid supply disruption	N/A	Variable but expedited (case specific)

Adapted from: FDA (2020) and FDA Press Announcement of March 31, 2020.

**Table 2:** European Medicines Agency (EMA) COVID-19 Pandemic Emergency Task Force response timelines.

Task or function	Response time to sponsor’s request	
	Typical	Emergency
Rapid scientific advice, at no cost to sponsors, without pre-established submission deadlines, more flexible requirements for scientific dossiers (i.e., IMPD)	40–70 days	20 days
Rapid agreement of pediatric investigation plans and rapid compliance check	120 days from first contact, 10 days for EMA decision following review	20 days (minimum), 2 days
Rolling review, which is an *ad hoc* procedure used in emergency contexts to allow the EMA to continuously assess the data for an upcoming highly promising application as they become available (i.e., preceding the formal submission of a complete application for a NMA).	N/A	Variable and case-specific (accelerated from normal cycle times)
Marketing authorization is expected to benefit from rolling review to minimize the common practice of stopping and re-starting the review clocks. Should an applicant not wish to use rolling review, or in case the application has not been accepted for such review, the applicant may still apply for accelerated assessment. In such case, the review of the application is started only after validation of a complete application.	210 days active review time	The maximum active review time is reduced to 150 days, which in practice may even be shorter, according to the EMA
Extension of indication and extension of marketing authorization. The abovementioned support measures are also available for already authorized products being repurposed for COVID-19	Variable (case specific)	Variable (case specific)
Compassionate use: certain unauthorized medicinal products may be made available at a national level through compassionate use programs during a health emergency to facilitate the availability of new experimental treatments that are still under development	Variable (case specific)	Variable (case specific)

Adapted from: EMA (2020b).

#### United States FDA Coronavirus Treatment Acceleration Program

In the spring of 2020, the FDA established a special emergency program for candidate diagnostics, vaccines, and therapies for SARS-CoV-2 and COVID-19. The Coronavirus Treatment Acceleration Program (CTAP)^5^ aims to utilize every available method to move new treatments to patients in need as quickly as possible, while simultaneously assessing the safety and efficacy of new modes of intervention. As of September 2020, CTAP was overseeing more than 300 active clinical trials for new treatments (>30 antivirals, >30 cell and gene therapies, >100 immunomodulators, >40 neutralizing antibodies, and >70 combination products and other categories) and was reviewing nearly 600 preclinical-stage programs for new medical interventions.

Responding to pressure for procedural streamlining and rapid response, the FDA refocused staff priorities, modified its guidelines to fit emergency situations, and achieved a remarkable set of benchmarks (Table 1). In comparison to the review and response timelines described in the previous section, the FDA’s emergency response structure within CTAP is exemplary and, as noted, these changes have successfully enabled the rapid evaluation of hundreds of new diagnostics and candidate vaccine and therapeutic products.

#### European Guidance for COVID-19 Medicine Developers and Companies

The European Medicines Agency has established initiatives for the provision of accelerated development support and evaluation procedures for COVID-19 treatments and vaccines. These initiatives generally follow the EMA Emergent Health Threats Plan published at the end of 2018 (EMA, 2018). Similar to FDA’s CTAP, EMA’s COVID-19 Pandemic Emergency Task Force (EMA, 2020b) aims to coordinate and enable fast regulatory action during the development, authorization, and safety monitoring of products or procedures intended for the treatment and prevention of COVID-19 (EMA, 2020a). Collectively, this task force and its accessory committees are empowered to rapidly address emergency use requests (Table 2). Although perhaps not as dramatic as the aspirational time reductions established by the FDA’s CTAP, the EMA’s refocusing of resources and shorter response times to accelerate the development and approval of emergency use products are nevertheless laudable. In the United Kingdom, the MHRA^6^ has also revised customary regulatory procedures to conform with COVID-19 emergency requirements by creating flexible regulations spanning early consultation, accelerated clinical development and review, and alternatives to facility inspection.

### Implications of Streamlined Regulations for the Development of PMP Emergency Response Diagnostics, Vaccines, Prophylactics, and Therapeutics

During a public health emergency, one can envision the preferential utilization of existing indoor (contained) manufacturing capacity, at least in the near term. Processes making use of indoor cultivation (whole plants or cell culture) and conventional purification can be scrutinized more quickly by regulatory agencies due to their familiarity, resulting in shorter time-to-clinic and time-to-deployment periods. Although many, perhaps most, process operations will be familiar to regulators, there are some peculiarities of plant-based systems that differentiate them from conventional processes and, hence, require the satisfaction of additional criteria. Meeting these criteria is in no way insurmountable, as evidenced by the rapid planning and implementation of PMP programs for SARS-CoV-2/COVID-19 by PMP companies such as Medicago, iBio, and Kentucky Bioprocessing.^7^

#### Rationale for the Choice of Expression Platform

During emergency situations when speed is critical, transient expression systems (Gleba et al., 2014; Hahn et al., 2015) are more likely to be used than stable transgenic hosts, unless GM lines were developed in advance and can be activated on the basis of demand (e.g., lines expressing interferons, broad-spectrum antiviral lectins, or anti-inflammatory proteins). The vectors used for transient expression in plants are non-pathogenic in mammalian hosts and environmentally containable if applied indoors, and by now they are well known to the regulatory agencies. Accordingly, transient expression systems have been deployed rapidly for the development of COVID-19 interventions.

The vaccine space has shown great innovation and the World Health Organization (WHO) has maintained a database of COVID-19 vaccines in development,^8^ including current efforts involving PMPs. For example, Medicago announced the development of its VLP-based vaccine against COVID-19 in March 2020, within 20 days of receiving the virus genome sequence, and initiated a Phase I safety and immunogenicity study in July.^9^ If successful, the company expects to commence Phase II/III pivotal trials by late 2020. Medicago is also developing therapeutic antibodies for patients infected with SARS-CoV-2, and this program is currently in preclinical development. Furthermore, iBio has announced the preclinical development of two SARS-CoV-2 vaccine candidates, one VLP and one subunit vaccine.^10^ Kentucky Bioprocessing has announced the production and preclinical evaluation of a conjugate TMV-based vaccine and has requested regulatory authorization for a first-in-human clinical study.^11^ These efforts required only a few months to reach these stages of development and are a testament to the rapid expression, prototyping, and production advantages offered by transient expression.

#### Regulatory Bias: Process vs. Product

The PMP vaccine candidates described above are all being developed by companies in North America. The rapid translation of PMPs from bench to clinic reflects the conformance of chemistry, manufacturing, and control (CMC) procedures on one hand, and environmental safety and containment practices on the other, with existing regulatory statutes. This legislative system has distinct advantages over the European model, by offering a more flexible platform for discovery, optimization, and manufacturing. New products are not evaluated for compliance with GM legislation as they are in the EU and the United States (Sparrow et al., 2013) but are judged on their own merits. In contrast, development programs in the EU face additional hurdles even when using well-known techniques and even additional scrutiny if new plant breeding technologies are used, such as the CRISPR/Cas9 system or zinc finger nucleases (Menary et al., 2020).

#### Manufacturing Process and Facility Validation

Process validation in manufacturing is a necessary but resource-intensive measure required for marketing authorization. Following the publication of the Guidance for Industry “Process Validation: General Principles and Practices,” and the EU’s revision of Annex 15 to Directive 2003/94/EC for medicinal products for human use and Directive 91/412/EEC for veterinary use, validation became a life-cycle process with three principal stages: (1) process design, (2) process qualification, and (3) continuous process verification (FDA, 2011; EMA, 2015, 2020c). During emergency situations, the regulatory agencies have authorized the *concurrent validation* of manufacturing processes, including design qualification (DQ), installation qualification (IQ), operational qualification (OQ), and performance qualification (PQ). Although new facility construction or repurposing/re-qualification may not immediately help with the current pandemic, given that only existing and qualified facilities will be used in the near term, it will position the industry for the rapid scale-up of countermeasures that may be applied over the next several years. An example is the April 2020 announcement by the Bill & Melinda Gates Foundation of its intention to fund “at-risk” development of vaccine manufacturing facilities to accommodate pandemic-relevant volumes of vaccines, before knowing which vaccines will succeed in clinical trials. Manufacturing at-risk with existing facilities is also being implemented on a global scale. The Serum Institute of India, the world’s largest vaccine manufacturer, is producing at-risk hundreds of millions of doses of the Oxford University COVID-19 vaccine, while the product is still undergoing clinical studies.^12^ Operation Warp Speed (OWS)^13^ in the United States is also an at-risk multi-agency program that aims to expand resources to deliver 300 million doses of safe and effective but “yet-to-be-identified” vaccines for COVID-19 by January 2021, as part of a broader strategy to accelerate the development, manufacturing, and distribution of COVID-19 countermeasures, including vaccines, therapeutics, and diagnostics. The program had access to US$10 billion initially and can be readily expanded. As of August 2020, OWS had invested more than US$8 billion in various companies to accelerate manufacturing, clinical evaluation, and enhanced distribution channels for critical products.^14^ For example, over a period of approximately 6 months, OWS helped to accelerate development, clinical evaluation (including Phase III pivotal studies), and at-risk manufacturing of two mRNA-based COVID-19 vaccines, with at least three more vaccines (including adenovirus-based and recombinant/baculovirus-based candidates) heading into advanced clinical development and large-scale manufacturing by September/October 2020.

At the time of writing, no PMP companies had received support from OWS. However, in March 2020, Medicago received CAD$7 million from the Government of Quebec (Medicago 2020c) and part of the Government of Canada CAD$192 million investment in expansion programs (Medicago, 2020d), both of which were applied to PMP vaccine and antibody programs within the company.^15^

#### Product Quality Attributes

Once manufactured, PMP products must pass quality criteria meeting a defined specification before they reach the clinic. These criteria apply to properties such as identity, uniformity, batch-to-batch consistency, potency, purity, stability (including API and the formation of aggregates, truncations, and low-molecular-weight species over time), residual DNA, absence of vector, low levels of plant metabolites such as pyridine alkaloids, and other criteria as specified in guidance documents (FDA, 2002; EMA, 2006). Host and process-related impurities in PMPs, such as residual HCP, residual vector, pyridine alkaloids from solanaceous hosts (e.g., nicotine, anabasine, and related alkaloids), phenolics, heavy metals (some of which can bioaccumulate in transfected plants), and other impurities that could introduce a health risk to consumers, have been successfully managed by upstream process controls and/or state-of-the-art purification methods and have not impeded the development of PMP products (Tusé, 2011; Ma et al., 2015).

The theoretical risk posed by non-mammalian glycans, once seen as the Achilles heel of PMPs, has not materialized in practice. Plant-derived vaccine antigens carrying plant-type glycans have not induced adverse events in clinical studies, where immune responses were directed primarily to the polypeptide portion of glycoproteins (McCormick et al., 2008; Tusé, 2011; Tusé et al., 2015). One solution for products intended for systemic administration, where glycan differences could introduce a pharmacokinetic and/or safety risk (such as mAbs or therapeutic enzymes), is the engineering of plant hosts to express glycoproteins with mammalian-compatible glycan structures (Strasser et al., 2004, 2014; Chen, 2016). For example, ZMapp (an antibody cocktail for the treatment of Ebola patients) was manufactured using the transgenic *N. benthamiana* line ΔXT/FT, expressing RNA interference constructs to knock down the expression of the enzymes XylT and FucT responsible for plant-specific glycans, as a chassis for transient expression of the mAbs (Hiatt et al., 2015).

In addition to meeting molecular identity and physicochemical quality attributes, PMP products must also be safe for use at the doses intended and efficacious in model systems *in vitro*, *in vivo*, and *ex vivo*, following the guidance documents listed above. Once proven efficacious and safe in clinical studies, successful biologic candidates can be approved *via* a BLA in the United States and a new marketing authorization (NMA) in the EU.

#### Deployment in Emergency Situations

In emergency situations, diagnostic reagents, vaccine antigens, and prophylactic and therapeutic proteins may be deployed prior to normal marketing authorization *via* fast-track procedures such as the FDA’s emergency use authorization (EUA).^16^ This applies to products approved for marketing in other indications that may be effective in a new emergency indication (repurposing), and new products that may have preclinical data but little or no clinical safety and efficacy data. Such pathways enable controlled emergency administration of a novel product to patients simultaneously with traditional regulatory procedures required for subsequent marketing approval.

In the United States, the FDA has granted EUAs for several diagnostic devices, personal protective devices, and certain other medical devices, and continuously monitors EUAs for drugs. For example, the EUA for chloroquine and hydroxychloroquine to treat COVID-19 patients was short-lived, whereas remdesivir remains under EUA evaluation for severe COVID-19 cases. The mRNA-based SARS-CoV-2 vaccines currently undergoing Phase III clinical evaluation by Pfizer/BioNTech and Moderna/NIAID, and other vaccines reaching advanced stages of development, are prime candidates for rapid deployment *via* the EUA process. No PMPs have yet been granted EUA, but plant-made antibodies and other prophylactic and therapeutic APIs may be evaluated and deployed *via* this route. One example of such a PMP candidate is griffithsin, a broad-spectrum antiviral lectin that could be administered as a prophylactic and/or therapeutic for viral infections, as discussed later.

The FDA’s EUA is a temporary authorization subject to constant review and can be rescinded or extended at any time based on empirical results and the overall emergency environment. Similarly, the EU has granted *conditional marketing authorisation* (12-month duration) to rapidly deploy drugs such as remdesivir for COVID-19 in parallel with the standard marketing approval process for the new indication.

#### Accelerated Product Development *via* the Animal Rule

The regulations commonly known as the *animal rule* (US 21 CFR 314.600-650 for drugs; 21 CFR 601.90-95 for biologics; first effective on 1 July 2002)^17^ allow for the approval of drugs and licensure of biologic products when human efficacy studies are not ethical and field trials to study the effectiveness of drugs or biologic products are not feasible. The animal rule is intended for drugs and biologics developed to reduce or prevent serious or life-threatening conditions caused by exposure to lethal or permanently disabling toxic chemical, biological, radiological, or nuclear substances. Under the animal rule, efficacy is established based on adequate and well-controlled studies in animal models of the human disease or condition of interest, and safety is evaluated under the pre-existing requirements for drugs and biologic products.

As an example, the plant-derived mAb cocktail ZMapp for Ebola virus disease, manufactured by Kentucky Bioprocessing for Mapp Biopharmaceutical (San Diego, CA, United States)^18^ and other partners, and deployed during the Ebola outbreak in West Africa in 2014, was evaluated only in primates infected with the Congolese variant of the virus (EBOV-K), with no randomized controlled clinical trial before administration to infected patients under a compassionate use protocol (Qiu et al., 2014). A conventional NIH-supported clinical study was conducted subsequent to first deployment (Davey et al., 2016).

#### Accelerated Product Development *via* Human Challenge Clinical Studies

Although the fast-track and streamlined review and authorization procedures described above can reduce time-to-deployment and time-to-approval for new or repurposed products, current clinical studies to demonstrate safety and efficacy generally follow traditional sequential designs. Products are licensed or approved for marketing based on statistically significant performance differences compared to controls, including placebo or standards of care, typically generated in large Phase III pivotal trials. One controversial proposal, described in a draft WHO report (World Health Organization Advisory Group, 2020), is to accelerate the assessment of safety and efficacy for emergency vaccines by administering the medical intervention with deliberate exposure of subjects to the threat agent in a challenge study.

Although the focus of the WHO draft report was on vaccines, the concept could conceivably be extended to non-vaccine prophylactics and therapeutics. Results could be generated quickly as the proportion of treated and control subjects would be known, as would the times of infection and challenge. Challenge studies in humans, also known as controlled human infection models or controlled human infection studies (CHIMs or CHIs, respectively), are fraught with ethical challenges but have already been used to assess vaccines for cholera, malaria, and typhoid (Cohen et al., 2002; Njue et al., 2018; Raymond et al., 2019). The dilemma for a pathogen like SARS-CoV-2 is that there is no rescue medication yet available for those who might contract the disease during the challenge, as there was for the other diseases, putting either study participants (due to current lack of effective treatment) or emergency staff (due to increased exposure) at risk (Shah et al., 2020).

### Perspective for PMP Regulatory Approval

In the EU, the current regulatory environment is a substantial barrier to the rapid expansion of PMP resources to accelerate the approval and deployment of products and reagents at relevant scales in emergency situations. A recent survey of the opinions of key stakeholders in two EU Horizon 2020 programs (Pharma-Factory and Newcotiana), discussing the barriers and facilitators of PMPs and new plant breeding techniques in Europe, indicated that the current (EU and United Kingdom) regulatory environment was seen as one of the main barriers to the further development and scale-up of PMP programs (Menary et al., 2020). In contrast, regulations have not presented a major barrier to PMP development in the United States or Canada, other than the lengthy timescales required for regulatory review and product approval in normal times.

Realizing current national and global needs, regulatory agencies in the United States, Canada, the EU, and the United Kingdom have drastically reduced the timelines for product review, conditional approval, and deployment. In turn, the multiple unmet needs for rapidly available medical interventions have created opportunities for PMP companies to address such needs with gene expression tools and manufacturing resources that they already possess. This has enabled the ultra-rapid translation of product concepts to clinical development in record times – weeks to months instead of months to years – in keeping with other high-performance biomanufacturing platforms. The current pandemic situation, plus the tangible possibility of global recurrences of similar threats, may provide an impetus for new investments in PMPs for the development and deployment of products that are urgently needed.

## Plant-Derived Products To Counteract Pandemics

### Considerations for SARS-CoV-2 Vaccines

An effective vaccine is the best long-term solution to COVID-19 and other pandemics. Worldwide, governments are trying to expedite the process of vaccine development by investing in research, testing, production, and distribution programs, and streamlining regulatory requirements to facilitate product approval and deployment and are doing so with highly aggressive timelines (Tables 1 and 2). A key question that has societal implications beyond vaccine development is whether the antibody response to SARS-CoV-2 will confer immunity against re-infection and, if so, for how long? Will humans who recover from this infection be protected against a future exposure to the same virus months or years later? Knowing the duration of the antibody response to SARS-CoV-2 vaccines will also help to determine whether, and how often, booster immunizations will be needed if the initial response exceeds the protection threshold (Moore and Klasse, 2020). It is clear that some candidate vaccines will have low efficacy (e.g., protection in <50% of individuals), some vaccines will have high efficacy (e.g., protection in 70–80% of individuals or more), and some will decline over time and will need booster doses.

An updated list of the vaccines in development can be found in the WHO draft landscape of COVID-19 candidate vaccines.^19^ As of August 2020, among the ~25 COVID vaccines in advanced development, five had entered Phase III clinical studies, led by Moderna/NIAID, Oxford University/Astra Zeneca, Pfizer/BioNTech, Sinopharm, and Sinova Biotech.^20^ Most of these candidates are intended to induce antibody responses that neutralize SARS-CoV-2, thereby preventing the virus from entering target cells and infecting the host. In some cases, the vaccines may also induce antibody and/or cellular immune responses that eliminate infected cells, thereby limiting the replication of the virus within the infected host (Moore and Klasse, 2020). The induction of neutralizing antibodies directed against the SARS-CoV-2 spike (S) glycoprotein (see Figures 1A,B in Moore and Klasse, 2020) is considered a priority. The immunogens used to elicit neutralizing antibodies are various forms of the S protein, including the isolated receptor-binding domain (RBD; Callaway, 2020; Quinlan et al., 2020). The S protein variants can be expressed *in vivo* from DNA or mRNA constructs or recombinant adenovirus or vaccinia virus vectors, among others. Alternatively, they can be delivered directly as recombinant proteins with or without an adjuvant or as a constituent of a killed virus vaccine (see Table 1 in Moore and Klasse, 2020). Many of these approaches are included among the hundreds of vaccine candidates now at the pre-clinical and animal model stages of development.

**Figure 1:**
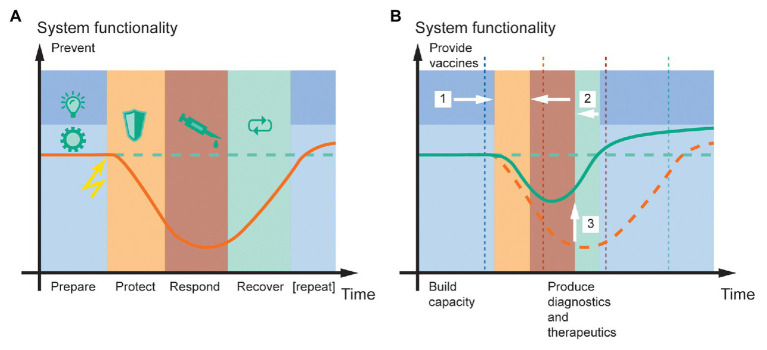
System functionality in the face of a pandemic, and the potential for resilience engineering based on molecular farming in plants. **(A)** The resilience cycle typically consists of five phases [*prevent* (dark blue), *prepare* (light blue), *protect* (orange), *respond* (red), and *recover* (green); Thoma et al., 2016]. Upon encountering a negative event (lightning symbol), the system loses functionality (orange line) compared to the pre-event state (dashed green line) until *protect* and *response* measures stabilize it at a certain level and *recover* measures can begin. **(B)** Plant molecular farming can improve public health resilience to pandemic disease outbreaks by (1) enabling the large-scale production of vaccines that reduce virus spreading and the likelihood of recurrent outbreaks, (2) facilitating faster response and recovery by rapidly providing diagnostics, emergency vaccines, and therapeutics, and (3) thereby minimizing the loss of system functionality (green line). A prerequisite to deliver these benefits is that sufficient production capacity is built before the event, during the *prepare* phase. For comparison, the original time points of phase transitions in **(A)** are shown as dashed vertical lines in **(B)**. The time and functionality scales are in arbitrary units but drawn to scale between panels **(A**,**B)**. The curves illustrate typical scenarios but are not quantitative.

Antibody responses in COVID-19 patients vary greatly. Nearly all infected people develop IgM, IgG, and IgA antibodies against the SARS-CoV-2 nucleocapsid (N) and S proteins 1–2 weeks after symptoms become apparent, and the antibody titers (sometimes including neutralizing antibodies) remain elevated for at least several weeks after the virus is no longer detected in the convalescent patient (Huang et al., 2020; Long et al., 2020; Ma et al., 2020; Okba et al., 2020). The nature and longevity of the antibody response to coronaviruses are relevant to the potency and duration of vaccine-induced immunity.

By far the most immunogenic vaccine candidates for antibody responses are recombinant proteins (Moore and Klasse, 2020). The most straightforward approach to vaccine development would be based on inactivated or attenuated strains of SARS-CoV-2, but the production of sufficient material generally takes longer than is the case for subunit vaccines, high-level containment would be necessary to grow the virus before attenuation/inactivation, and the candidates would carry a risk of reacquired virulence (Regla-Nava et al., 2015). For subunit vaccines, target antigens must be selected carefully. Research on the original SARS-CoV strain indicated that the N protein is highly conserved among coronavirus families, including strains responsible for mild respiratory tract infections, thus suggesting the possibility of developing a universal vaccine. However, antibodies induced by N proteins did not provide protective immunity; likewise, the M and E proteins elicited only weak protective responses (Gralinski and Menachery, 2020). These studies helped to confirm the S protein as the most suitable target for eliciting a neutralizing humoral response.

### Potential for Plant-Produced Vaccines

The entry of coronaviruses into host cells is facilitated by the S protein, which assembles into homotrimers on the virus surface (Tortorici and Veesler, 2019). The S protein comprises two functional subunits: S1, which binds to the host cell receptor, and S2, which facilitates the fusion of the viral and host cell membranes. For many coronaviruses, the S protein is cleaved at the boundary between the S1 and S2 subunits and mostly remains non-covalently bound in the pre-fusion conformation (Kirchdoerfer et al., 2018). Hence, the uptake of coronaviruses into host cells is a complex process that requires receptor binding and proteolytic processing of the S protein to stimulate membrane fusion and viral uptake (Walls et al., 2020).

Companies currently developing COVID-19 vaccines are mainly expressing variants of the SARS-CoV-2 S1 protein or RBD. The S1 proteins of SARS-CoV and SARS-CoV-2 are heavily glycosylated, with an approximately equal mixture of complex and high-mannose glycans (Shajahan et al., 2020; Watanabe et al., 2020). It is unclear whether plant-type complex glycans would affect the efficacy of a recombinant SARS-CoV-2 S-protein vaccine expressed in plants. High-mannose glycans are generally conserved across higher eukaryotes, so it could be expected that at least some high-mannose glycans will be added during the expression of the antigen in plants. Furthermore, it is not clear whether sialic acid plays a role in host-receptor interactions. This is not generally present on native or recombinant plant glycoproteins, although engineered plant varieties that produce sialylated proteins have been described (Kallolimath et al., 2016).

Virus-like particles displaying SARS-CoV-2 antigens are larger than subunit vaccines, promoting recognition and internalization by antigen-presenting cells and thus triggering an adaptive immune response. Furthermore, the regular array of epitopes acts as pathogen-associated molecular patterns to induce strong cellular and humoral responses (Lua et al., 2014). VLPs are readily produced at scale in plants by molecular farming (Rybicki, 2017). The Medicago VLP platform is a prime example and has previously been used to produce millions of doses of seasonal influenza vaccines (D’Aoust et al., 2010; Wu, 2020). Furthermore, iBio is also using a proprietary system to develop VLP-based vaccines in *N. benthamiana* plants.

### Production of Therapeutic Proteins in Plants

Given the time required to develop and test a COVID-19 vaccine, the possibility that a vaccine may not be effective in all populations due to the variability of immune responses, and the likelihood that SARS-CoV-2 will mutate, we foresee an ongoing demand for therapeutic proteins, such as mAbs, immunoadhesins, interferons, and antivirals, to either target the virus itself or reduce the severity of the associated acute respiratory syndrome (Capell et al., 2020; Rosales-Mendoza, 2020).

#### Monoclonal Antibodies

Several recombinant mAbs and antibody cocktails against COVID-19 are currently undergoing clinical development for therapeutic and prophylactic applications, including REGN-CoV-2 (Regeneron Therapeutics, Phase III), CSL312 (CSL Behring, Phase II), LY-CoV555 (Eli Lilly/AbCellera, Phase III), and TYO27 (Tychan, Phase I; Marovich et al., 2020). Many of the mAbs in development target the S-protein, aiming to block interactions with its receptor, angiotensin-converting enzyme 2 (ACE2). Efforts to exploit convalescent sera from patients who recovered from COVID-19 have helped identify antibodies with neutralizing potential. For example, Eli Lilly/AbCellera identified such an antibody in a blood sample from one of the first United States patients who recovered from the disease. The mAb was developed into LY-CoV555, a potent, neutralizing IgG1 that binds the S protein. In collaboration with NIAID, the product began Phase III clinical evaluation in high-risk assisted living facilities in August 2020.^21^

Most COVID-19 antibody products in development are produced in mammalian cells, but antibodies were among the first products of molecular farming in plants (Hiatt et al., 1989) and many different mAb products have been expressed, including complex secretory IgA (Wycoff, 2005). The dose of a mAb or mAb cocktail needed for the prevention or treatment of COVID-19 is currently unclear. About 9 g of the ZMapp cocktail was needed per treatment against Ebola virus and in a subsequent clinical study (Davey et al., 2016), but that dose level was selected from the outcome of studies in non-human primates (animal rule), which enabled rapid deployment under the compassionate use protocol and did not benefit from dose optimization studies in humans. Assuming similar doses, manufacturing scalability is likely to be a key challenge in the production of COVID-19 antibodies. The scaling up of conventional bioreactors is particularly challenging due to changes in mixing, mass transfer, and heat exchange, whereas transient expression in plants can be scaled in a linear manner because each plant is effectively an independent bioreactor, equating to a process of numbering up by increasing the plant inventory and throughput of the facility. Similarly, cost will be an important consideration. In 2013, total sales of mAbs produced in mammalian cell bioreactors amounted to ~€48.5 (US$57) billion for 8,182 kg of product, with an average sales price of ~€5,957 ($7,000) g^−1^ (Ecker et al., 2015). Production costs and capital expenses for the transient expression of mAbs in plants are estimated to be at least 50% lower than mammalian cell culture production facilities (Nandi et al., 2016), allowing manufacturers to reduce sales prices while still making some profit or providing these therapeutics at cost, and saving taxpayer resources.

#### Immunoadhesins

Another promising therapeutic approach is the use of plants to produce immunoadhesins (Wycoff et al., 2015). Such molecules combine the virus-binding region of a receptor, in this case ACE2, with the immunoglobulin Fc domain (Kruse, 2020; Qian and Hu, 2020). The ACE2 component acts as a decoy to bind SARS-CoV-2 *via* the S protein, preventing it from engaging with native ACE2 on the surface of human cells, while the Fc region confers a longer circulatory half-life and provides effector functions that promote viral clearance, as well as facilitating product purification by Protein A affinity chromatography during manufacturing. Immunoadhesins form dimers *via* disulfide linkages between Fc domains, increasing their avidity when binding the S protein. One advantage of this strategy is that if the coronavirus mutates to escape binding to the immunoadhesins, it would similarly lose affinity for native ACE2, reducing its infectivity. Likewise, the SARS virus that re-emerged in 2003–2004 had a lower affinity for ACE2 than the original isolate, resulting in less severe infections and no secondary transmission (Li et al., 2005). An additional advantage of this strategy is that exogenous ACE2 would compensate for lower ACE2 levels in the lungs during infection, thereby contributing to the treatment of acute respiratory distress. Several companies in the United States and the EU have developed recombinant ACE2 and ACE2-Fc fusion proteins for preclinical and clinical testing, although all these products are currently produced in mammalian cell lines (Qian and Hu, 2020). The impact of plant-specific complex glycans on the ability of ACE2-Fc to bind the RBD has been studied using molecular dynamic simulations and illustrates the important role that glycosylation may play in the interaction between the S protein and ACE2 (Bernardi et al., 2020).

#### Broad-Spectrum Antiviral Griffithsin

Griffithsin is a lectin that binds high-mannose glycans, and is currently undergoing clinical development as an antiviral against HIV-1. However, it also binds many other viruses that are pathogenic in humans, including HSV (Nixon et al., 2013), HCV (Meuleman et al., 2011), Nipah virus (Lo et al., 2020), Ebola virus, and coronaviruses including SARS-CoV and MERS (O’Keefe et al., 2010), and as recently determined, also SARS-CoV-2. A clinical product in development by University of Louisville is currently manufactured in *N. benthamiana* by Kentucky Bioprocessing using a TMV vector. The API is also undergoing preclinical development as a nasal spray for use as a non-vaccine prophylactic against coronaviruses, with clinical evaluation planned for 2020 (University of Pittsburgh, 2020). This candidate PMP antiviral could be deployed under the EUA pathway if found effective in controlled clinical studies.

Griffithsin is an interesting example of a product that is ideally matched to plant-based manufacturing because it is naturally produced by a marine alga. Griffithsin has been expressed with limited success in *E. coli* and tobacco chloroplasts, but better results have been achieved by transient expression in *N. benthamiana* using *A. tumefaciens* infiltration or TMV vectors, with expression levels of up to 1 g kg^−1^ fresh mass and recoveries of up to 90% (Vafaee et al., 2014; Fuqua et al., 2015a,b; Hahn et al., 2015). A TEA model of griffithsin manufactured in plants at initial commercial launch volumes (20 kg) for use in HIV microbicides revealed that process was readily scalable and (subject to efficiency improvements) could provide the needed market volumes of the lectin within an acceptable range of costs, even for cost-constrained markets (Alam et al., 2018). The manufacturing process was also assessed for environmental, health, and safety impact and found to have a highly favorable environmental output index with negligible risks to health and safety.

#### Production of Diagnostic Reagents in Plants

In addition to COVID-19 PCR tests, which detect the presence of SARS-CoV-2 RNA, there is a critical need for protein-based diagnostic reagents that test for the presence of viral proteins and thus report a current infection, as well as serological testing for SARS-CoV-2 antibodies that would indicate prior exposure, recovery, and possibly protection from subsequent infection. The most common formats for these tests are the ELISA and lateral flow assay. The design and quality of the binding reagents (antibodies to SARS-CoV-2 proteins for the viral antigen tests, or full-length/truncated SARS-CoV-2 proteins for the serological tests), along with other test conditions such as sample quality, play a key role in establishing the test specificity and selectivity, which determine the proportion of false positive and false negative results. Although the recombinant protein mass needed for diagnostic testing is relatively small (0.3–1.0 μg per test), the number of tests needed for the global population is massive, given that many individuals will need multiple and/or frequent tests. For example, 8 billion tests would require a total of ~2.5 kg purified recombinant protein, which is not an insurmountable target. However, although the production of soluble trimeric full-length S protein (as a diagnostic reagent for the serological test) by transient transfection in HEK293 cells has been improved by process optimization, current titers are only ~5 mg L^−1^ after 92 h (Esposito et al., 2020). Given a theoretical recovery of 50% during purification, a fermentation volume of 1,000 m^3^ would be required to meet the demand for 2.5 kg of this product. Furthermore, to our knowledge, the transient transfection of mammalian cells has only been scaled up to ~0.1 m^3^ (Girard et al., 2002). The transient expression of such protein-based diagnostic reagents in plants could increase productivity while offering lower costs and more flexibility to meet fluctuating demands or the need for variant products. Furthermore, diagnostic reagents can include purification tags with no safety restrictions, and quality criteria are less stringent compared to an injectable vaccine or therapeutic. Several companies have risen to the challenge of producing such reagents in plants, including Diamante (Verona, Italy), Leaf Expression Systems (Norwich, United Kingdom), and a collaborative venture between PlantForm, Cape Bio Pharms, Inno-3B, and Microbix.

### Targeting the Resilience Cycle With Plant Molecular Farming

Resilience is the state of preparedness of a system, defining its ability to withstand unexpected, disastrous events (such as outbreaks of pandemic disease), and to preserve critical functionality while responding quickly so that normal functionality can be restored (Thoma et al., 2016). The concept was popularized by the 2011 Fukushima nuclear accident (Hollnagel and Fujita, 2013) but received little attention in the pharmaceutical sector until COVID-19. Of the 277 publications retrieved from the National Library of Medicine^22^ on July 9th 2020 using the search terms “resilience” and “pandemic,” 82 were evenly distributed between 2002 and 2019 (~5 per year) and 195 were published between January and July 2020.

Resilience can be analyzed by defining up to five stages of a resilient system under stress, namely *prevent* (optional), *prepare*, *protect*, *respond*, and *recover* (Figure 1A; Thoma et al., 2016). Here, *prevent* includes all measures to avoid the problem all together. In the context of COVID-19, this may have involved the banning of bush meat from markets in densely populated areas (Li et al., 2019). The *prepare* stage summarizes activities that build capacities to protect a system and pre-empt a disruptive event. In a pandemic scenario, this can include stockpiling personal protective equipment but also ensuring the availability of rapid-response biopharmaceutical manufacturing capacity. The *protect* and *respond* stages involve measures that limit the loss of system functionality (e.g., emergency hospitalization capacity or gross domestic product) and minimize the time until it starts to recover, respectively. In terms of a disease outbreak, the former can consist of quarantining infected persons, especially in the healthcare sector, to avoid super-spreaders and maintain healthcare system operability (Steiner et al., 2020). The *response* measures may include passive strategies such as the adjustment of legislation, including social distancing and public testing regimes, or active steps such as the development of vaccines and therapeutics (Grein et al., 2020). Finally, the *recover* phase is characterized by regained functionality, for example by reducing the *protect* and *response* measures that limit system functionality, such as production lockdown. Ultimately, this can result in an increased overall system functionality at the end of a resilience cycle and before the start of the next “iteration” (Figure 1B). For example, a system such as society can be better prepared for a pandemic situation due to increased pharmaceutical production capacity or platforms like plants.

From our perspective, the production of recombinant proteins in plants could support the engineering of increased resilience primarily during the *prepare* and *respond* stages and, to a lesser extent, during the *prevent* and *recover* stages (Figure 1B). During the *prepare* stage, it is important to build sufficient global production capacity for recombinant proteins to mount a rapid and scalable response to a pandemic. These capacities can then be used during the *response* stage to produce appropriate quantities of recombinant protein for diagnostic (antigens and mAbs), prophylactic (vaccines or lectins), or therapeutic (mAbs) purposes as discussed above. The speed of the plant system will reduce the time taken to launch the *response* and *recovery* stages, and the higher the production capacity, the more system functionality can be maintained. The same capacities can also be used for the large-scale production of vaccines in transgenic plants if the corresponding pathogen has conserved antigens. This would support the *prevent* stage by ensuring a large portion of the global population can be supplied with safe and low-cost vaccines, for example, to avoid recurrent outbreaks of the disease. Similarly, existing agricultural capacities may be re-directed to pharmaceutical production as recently discussed (Webb et al., 2020). There will be indirect benefits during the *recover* phase because the speed of plant-based production systems will allow the earlier implementation of measures that bring system functionality back to normal, or at least to a “new or next normal.” Therefore, we conclude that plant-based production systems can contribute substantially to the resilience of public healthcare systems in the context of an emergency pandemic.

## Production Cost and Global Capacity of Plant-Based Systems

### Product-Dependent and Process-Dependent Costs

The cost of pharmaceuticals is increasing in the United States at the global rate of inflation, and a large part of the world’s population cannot afford the cost of medicines produced in developed nations^23^ (Wineinger et al., 2019). Technical advances that reduce the costs of production and help to ensure that medicines remain accessible, especially to developing nations, are, therefore, welcome. Healthcare in the developing world is tied directly to social and political will, or the extent of government engagement in the execution of healthcare agendas and policies (Hefferon, 2014). Specifically, community-based bodies are the primary enforcers of government programs and policies to improve the health of the local population (Langridge, 2012; Tsekoa et al., 2020).

Planning for the expansion of a biopharmaceutical manufacturing program to ensure that sufficient product will be available to satisfy the projected market demand should ideally begin during the early stages of product development. Efficient planning facilitates reductions in the cost and time of the overall development process to shorten the time to market, enabling faster recouping of the R&D investment and subsequent profitability. In addition to the cost of the API, the final product form (e.g., injectable vs. oral formulation), the length and complexity of the clinical program for any given indication (e.g., infectious disease vs. oncology), and the course of therapy (e.g., vaccination vs. chronic care) have a major impact on cost. The cost of a pharmaceutical product, therefore, depends on multiple economic factors that ultimately shape how a product’s sales price is determined (Azhakanandam et al., 2015). Product-dependent costs and pricing are common to all products regardless of platform.

Plant-based systems offer several options in terms of equipment and the scheduling of upstream production and DSP, including their integration and synchronization (Spiegel et al., 2019). Early process analysis is necessary to translate R&D methods into manufacturing processes (Nandi et al., 2005). The efficiency of this translation has a substantial impact on costs, particularly if processes are frozen during early clinical development and must be changed at a subsequent stage. Process-dependent costs begin with production of the API. The manufacturing costs for PMPs are determined by upstream (biomass) production and downstream recovery and purification costs. The cost of biopharmaceutical manufacturing depends mostly on protein accumulation levels, the overall process yield, and the production scale.

Techno-economic assessment models for the manufacture of biopharmaceuticals are rarely presented in detail, but analysis of the small number of available PMP studies (Nandi et al., 2005, 2016; Buyel and Fischer, 2012; Tusé et al., 2014; Walwyn et al., 2015; Alam et al., 2018; Corbin et al., 2020) has shown that the production of biopharmaceuticals in plants can be economically more attractive than in other platforms (Nandi et al., 2016; Gengenbach et al., 2019; Corbin et al., 2020). A simplified TEA model was recently proposed for the manufacture of mAbs using different systems, and this can be applied to any production platform, at least in principle, by focusing on the universal factors that determine the cost and efficiency of bulk drug manufacturing (Mir-Artigues et al., 2019).

Minimal processing may be sufficient for oral vaccines and some environmental detection applications and can thus help to limit process development time and production costs (Rosenberg et al., 2015). However, most APIs produced in plants are subject to the same stringent regulation as other biologics, even in an emergency pandemic scenario (see section “Regulatory considerations for product approval and deployment during public health emergency situations”). It is, therefore, important to balance production costs with potential delays in approval that can result from the use of certain process steps or techniques. For example, flocculants can reduce consumables costs during clarification by 50% (Buyel and Fischer, 2014b), but the flocculants that have been tested are not yet approved for use in pharmaceutical manufacturing. Similarly, elastin-like peptides and other fusion tags can reduce the number of unit operations in a purification process, streamlining development and production, but only a few are approved for clinical applications (Jin et al., 2017). At an early pandemic response stage, speed is likely to be more important than cost, and production will, therefore, rely on well-characterized unit operations that avoid the need for process additives such as flocculants. Single-use equipment is also likely to be favored under these circumstances, because although more expensive than permanent stainless-steel equipment, it is also more flexible (modules of different sizes can be integrated as required) and there is no need for cleaning or cleaning validation between batches or campaigns, allowing rapid switching to new product variants if required. As the situation matures (and the production scale increases), a shift toward cost-saving operations and multi-use equipment would be more beneficial.

### Capacity Requirements for an Effective Global Response to SARS-CoV-2

An important question is whether current countermeasure production capacity is sufficient to meet the needs for COVID-19 therapeutics, vaccines, and diagnostics. For example, a recent report from the Duke Margolis Center for Health Policy^24^ estimated that ~22 million doses of therapeutic mAbs would be required to meet demand in the United States alone (including non-hospitalized symptomatic patients, hospitalized patients, and people in the same household as those who contract COVID-19), assuming one dose per patient and using rates of infection estimated in June 2020. The current demand for non-COVID-19 mAbs in the United States is >50 million doses per year^27^, so COVID-19 has triggered a 44% increase in demand in terms of doses. Although the mAb doses required for pre-exposure and post-exposure COVID-19 treatment will not be known until the completion of clinical trials, it is likely to be 1–10 g per patient based on the dose ranges being tested and experience from other disease outbreaks such as Ebola (Davey et al., 2016). Accordingly, 22–222 tons of mAb would be needed per year, just in the United States. The population of the United States represents ~4.25% of the world’s population, suggesting that 500–5,200 tons of mAb would be needed to meet global demand. The combined capacity of mammalian cell bioreactors (mainly in North America, Europe, and Asia) is ~6 million liters^27^, and even assuming mAb titers of 2.2 g L^−1^, which is the mean titer for well-optimized large-scale commercial bioreactors (Budzinski et al., 2019), a 13-day fed-batch culture cycle (28 batches per year), and a 30% loss in downstream recovery, the entirety of global mammalian cell bioreactor capacity could only provide ~259 tons of mAb per year. In other words, if the mammalian cell bioreactors all over the world were repurposed for COVID-19 mAb production, it would be enough to provide treatments for 50% of the global population if low doses (1 g or lower) were effective but only 5% if high doses (~10 g) were required. This illustrates the importance of identifying mAbs that are effective at the lowest dose possible, production systems that can achieve high titers and efficient downstream recovery, and the need for additional production platforms that can be mobilized quickly and that *do not rely* on bioreactor capacity. Furthermore, it is not clear how much of the existing bioreactor capacity can be repurposed quickly to satisfy pandemic needs, considering that ~78% of that capacity is dedicated to in-house products, many to treat cancer and other life-threatening diseases (Rader and Langer, 2018). The demand-on-capacity for vaccines will fare better, given the amount of protein per dose is 1 × 10^4^ to 1 × 10^6^ times lower than a therapeutic mAb. Even so, most of the global population (~7.8 billon people) may need to be vaccinated against SARS-CoV-2 over the next 2–3 years to eradicate the disease, and it is unclear whether sufficient quantities of vaccine can be made available, even if using adjuvants to reduce immunogen dose levels and/or the number of administrations required to induce protection. Even if an effective vaccine or therapeutic is identified, it may be challenging to manufacture and distribute this product at the scale required to immunize or treat most of the world’s population (Hosangadi et al., 2020; Zerhouni et al., 2020). In addition, booster immunizations, viral antigen drift necessitating immunogen revision/optimization, adjuvant availability, and standard losses during storage, transport, and deployment may still make it difficult to close the supply gap.

Regardless of the product, the supply of recombinant proteins is challenging during emergency situations due to the simultaneous requirements for rapid manufacturing and extremely high numbers of doses. The realities we must address include: (1) the projected demand exceeds the entire manufacturing capacity of today’s pharmaceutical industry (even if the production of all other biologics is paused); (2) there is a shortage of delivery devices (syringes) and the means to fill them; (3) there is insufficient lyophilization capacity to produce dry powder for distribution; and (4) distribution, including transportation and vaccination itself, will be problematic on such a large scale without radical changes in the public health systems of most countries. Vaccines developed by a given country will almost certainly be distributed within that country and to its allies/neighbors first and, thereafter, to countries willing to pay for priority. One solution to the product access challenge is to decentralize the production of countermeasures, and in fact one of the advantages of plant-based manufacturing is that it decouples developing countries from their reliance on the pharmaceutical infrastructure. Hence, local production facilities could be set up based on greenhouses linked to portable clean rooms housing disposable DSP equipment. In this scenario, the availability of multiple technology platforms, including plant-based production, can only be beneficial.

### Impact of IP on Freedom to Operate for Rapid Manufacturing of Critical Supplies

Several approaches can be used to manage potential IP conflicts in public health emergencies that require the rapid production of urgently needed products. Licensing (including cross-licensing) of key IP to ensure freedom to operate (FTO) is preferred because such agreements are cooperative rather than competitive. Likewise, cooperative agreements to jointly develop products with mutually beneficial exit points offer another avenue for productive exploitation. These arrangements allow collaborating institutions to work toward a greater good.

Licensing has been practiced in past emergencies when PMP products were developed and produced using technologies owned by multiple parties. In the authors’ experience, the ZMapp cocktail (deployed in the 2014 outbreak of Ebola virus) was subject to IP ownership by multiple parties covering the compositions, the gene expression system, manufacturing process technology/knowhow, and product end-use. Stakeholders included the Public Health Agency of Canada’s National Microbiology Laboratory, the United States Army Medical Research Institute of Infectious Diseases (USAMRIID), Mapp Biopharmaceutical, Icon Genetics, and Kentucky Bioprocessing, among others. Kentucky Bioprocessing is also involved in a more recent collaboration to develop a SARS-CoV-2 vaccine candidate, aiming to produce 1–3 million doses of the antigen, with other stakeholders invited to take on the tasks of large-scale antigen conjugation to the viral delivery vector, product fill, and clinical development.^25^

Collaboration and pooling of resources and knowhow among big pharma/biopharma companies raises concerns over antitrust violations, which could lead to price fixing and other unfair business practices. With assistance from the United States Department of Justice (DOJ), this hurdle has been temporarily overcome by permitting several biopharma companies to share knowhow around manufacturing facilities and other information that could accelerate the manufacturing of COVID-19 mAb products.^26^ Genentech (United States subsidiary of Roche), Amgen, AstraZeneca, Eli Lilly, GlaxoSmithKline, and AbCellera Biologics will share information about manufacturing facilities, capacity, raw materials, and supplies in order to accelerate the production of mAbs even before the products gain regulatory approval. This is driven by the realization that none of these companies can satisfy more than a small fraction of projected demands by acting alone. Under the terms imposed by the DOJ, the companies are not allowed to exchange information about manufacturing cost of goods or sales prices of their drugs, and the duration of the collaboration is limited to the current pandemic.

Yet another approach is a government-led strategy in which government bodies define a time-critical national security need that can only be addressed by sequestering critical technology (including IP, reagents, materials, software, facilities, knowhow, and existing stockpiles) controlled by the private sector. In the United States, for example, the Defense Production Act was first implemented in 1950 but has been reauthorized more than 50 times since then (FEMA, 2009). Similar national security directives exist in Canada and the EU. In the United States, the Defense Production Act gives the executive branch substantial powers, allowing the president, largely through executive order, to direct private companies to prioritize orders from the federal government. The president is also empowered to “allocate materials, services, and facilities” for national defense purposes. The Defense Production Act has been implemented during the COVID-19 crisis to accelerate manufacturing and the provision of medical devices and personal protective equipment, as well as drug intermediates.

Therefore, a two-tiered mechanism exists to create FTO and secure critical supplies: the first and more preferable involving cooperative licensing/cross-licensing agreements and manufacturing alliances, and alternatively (or if the first should fail), a second mechanism involving legislative directives.

## Conclusion: Advantages of Plant Molecular Farming as a First Response to Global Pandemics

Many companies have modified their production processes to manufacture urgently-required products in response to COVID-19, including distillers and perfume makers switching to sanitizing gels, textiles companies making medical gowns and face masks, and electronics companies making respirators.^27^ Although this involves some challenges, such as production safety and quality requirements, it is far easier than the production of APIs, where the strict regulations discussed earlier in this article must be followed. The development of a mammalian cell line achieving titers in the 5 g L^−1^ range often takes 10–12 months or at least 5–6 months during a pandemic (Kelley, 2020). These titers can often be achieved for mAbs due to the similar properties of different mAb products and the standardized DSP unit operations (Gottschalk, 2016), but the titers of other biologics are often lower due to product toxicity or the need for bespoke purification strategies. Even if developmental obstacles are overcome, pharmaceutical companies may not be able to switch rapidly to new products because existing capacity is devoted to the manufacture of other important biopharmaceuticals. The capacity of mammalian cell culture facilities currently exceeds market demand by ~30% (Ecker and Seymour, 2020). Furthermore, contract manufacturing organizations (CMOs), which can respond most quickly to a demand for new products due to their flexible business model, control only ~19% of that capacity. From our experience, this CMO capacity is often booked in advance for several months if not years, and little is available for short-term campaigns. Furthermore, even if capacity is available, the staff and consumables must be available too. Finally, there is a substantial imbalance in the global distribution of mammalian cell culture capacity, favoring North America and Europe. This concentration is risky from a global response perspective because these regions were the most severely affected during the early and middle stages of the COVID-19 pandemic, and it is, therefore, possible that this capacity would become unusable following the outbreak of a more destructive virus.

**Figure 2:**
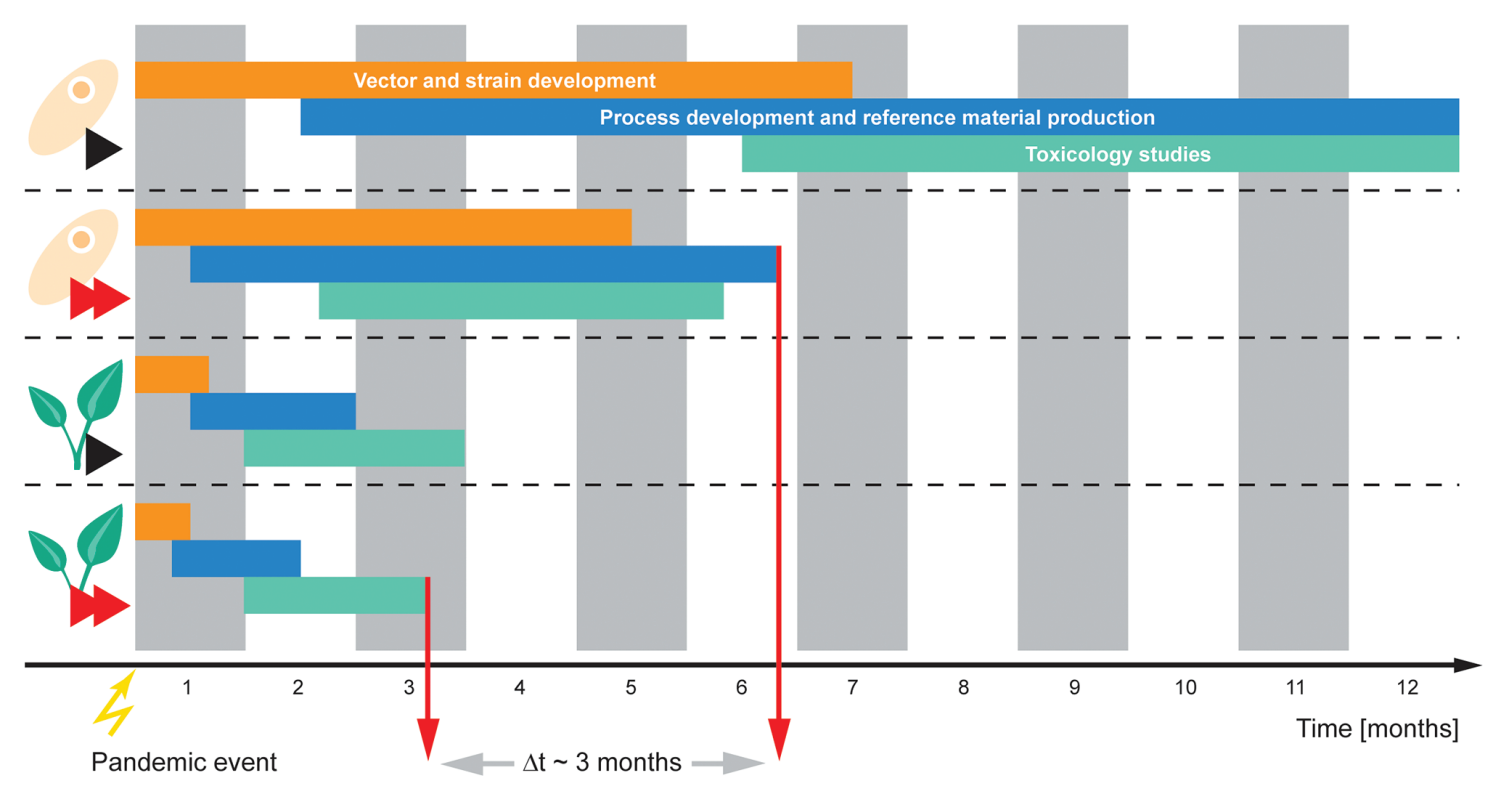
Comparison of mammalian cell culture and transient expression in plants for the production of emergency biopharmaceuticals. Timelines for conventional scheduling (black arrows) and accelerated procedures (double red arrows) are based on recent publications and announcements, as well as the authors’ experience (Shoji et al., 2011, 2012; Kelley, 2020). Transient expression allows much quicker vector development, process development, and reference material production, whereas the duration of toxicity studies is not reduced to the same degree because the time needed to run the studies remains the same regardless of the platform. Even so, transient expression in plants has the potential to reduce the emergency response time from gene sequence to clinical trial by at least 50% from ~6 months to <3 months.

Patents covering several technologies related to transient expression in plants will end during or shortly after 2020, facilitating the broader commercial adoption of the technology. This could accelerate the development of new PMP products in a pandemic situation (see section “Plant-derived products to counteract pandemics”). However, PMP production capacity is currently limited. There are less than five large scale PMP facilities in operation, and we estimate that these facilities could manufacture ~2,200 kg of product per year, assuming a combined annual biomass output of ~1,100 tons as well as similar recombinant protein production (~2 g kg^−1^) and DSP losses (30%) as for mammalian cells. Therefore, plant-based production certainly does currently not meet the anticipated demand for pandemic countermeasures. We have estimated a global demand of 500–5,200 tons per year for mAbs, depending on the dose, but only ~259 tons per year can be produced by using the current global capacity provided by mammalian cell bioreactors (at least based on publicly-available data) and plant-based systems currently represent less than 1% of the global production capacity of mammalian cell bioreactors. Furthermore, the number of plant molecular farming companies decreased from 37 to 23 between 2005 and 2020, including many large industry players that would be most able to fund further technology development (Fischer and Buyel, 2020). Nevertheless, the current plant molecular farming landscape has three advantages in terms of a global first-line response compared to mammalian cells. First, almost two thirds of global production capacity is held by CMOs or hybrid companies (working as CMOs while pursuing their own product pipeline), which can make their facilities available for production campaigns on short notice, as shown by their rapid response to COVID-19 allowing most to produce initial product batches by March 2020. In contrast, only ~20% of fermentation facilities are operated by CMOs (Seymour and Ecker, 2017). Second, despite the small number of plant molecular farming facilities, they are distributed around the globe with sites in the United States, Canada, United Kingdom, Germany, Japan, Korea, and South Africa, with more planned or under construction in Brazil and China (the largest facilities are currently located in North America and Europe). Finally, transient expression in plants is much faster than any other eukaryotic system with a comparable production scale, moving from gene to product within 20 days and allowing the production of up to 7,000 kg biomass per batch with product accumulation of up to 2 g kg^−1^ (Holtz et al., 2015; Zischewski et al., 2015). Even if the time required for protein production in mammalian cells can be reduced to 6 months as recently proposed (Kelley, 2020), Medicago has shown that transient expression in plants can achieve the same goals in less than 3 months (Figure 2). Therefore, the production of vaccines, therapeutics, and diagnostics in plants has the potential to function as a first line of defense against pandemics. Given the limited number and size of plant molecular farming facilities, we believe that the substantial investments currently being allocated to the building of biopharmaceutical production capacity should be shared with PMP production sites, allowing this technology to be developed as another strategy to improve our response to future pandemics.

## Author Contributions

DT, SN, KAM, and JB jointly wrote the manuscript. JB combined the contributions, revised the text, and prepared the figures. All authors contributed to the article and approved the submitted version.

## Disclaimer

The literature on COVID-19 accumulates daily, and we have cited not only peer-reviewed publications but also manuscripts deposited on preprint servers and reliable online sources. We acknowledge that some information in such sources may be inaccurate or outdated by the time this manuscript is published. As such, we urge readers to inspect key papers themselves and also recommend the use of additional resources to reach conclusions. We have no doubt that we may have overlooked some key papers in this rapidly evolving area of research. In addition, some information described herein was based on the authors’ personal participation in R&D projects or their direct knowledge of events, and as such no citable references existed at the time of writing. We have noted such instances in the text as “*authors’ experience*.”

### Conflict of Interest

DT was employed by DT/Consulting Group and GROW Biomedicine LLC. All authors declare that the research was conducted in the absence of any commercial or financial relationships that could be construed as a potential conflict of interest.

## Figures and Tables

**Table:** 

Term	Definition
API	Active pharmaceutical ingredient
BLA	Biologics License Application
BSL	Biosafety level
CHMP	Committee for Medicinal Products for Human Use
cGMP	Current good manufacturing practice
CTA	Clinical Trial Application
CV	Coefficient of variation
EMA	European Medicines Agency
EOP	End of phase
FDA	Food and Drug Administration
EUA	Emergency use authorization
GMO	Genetically modified organisms
HCP	Host cell proteins
ICH	International Council for Harmonization
IMPD	Investigational Medicinal Product Dossier
IND	Investigational New Drug
IP	Intellectual property
mAbs	Monoclonal antibodies
MERS	Middle East Respiratory Syndrome
NDA	New Drug Application
NMA	New marketing authorization
PMP	Plant-made pharmaceuticals
R&D	Research and development
SARS	Severe acute respiratory syndrome
T-DNA	Transferred DNA
TEA	Techno-economic assessment
TMV	Tobacco mosaic virus
VLP	Virus-like particles

## Footnotes

^1^https://coronavirus.jhu.edu/map.html

^2^International Council for Harmonization of Technical Requirements for Pharmaceuticals for Human Use (ICH) website. Accessed July 14, 2020. https://www.ich.org/

^3^European Investigational Medicinal Product Dossiers. Accessed July 14, 2020. http://www.imp-dossier.eu/

^4^USDA/APHIS SECURE Rule revision to 7 CFR 340. Accessed July 17, 2020. https://www.aphis.usda.gov/aphis/ourfocus/biotechnology/biotech-rule-revision/secure-rule/secure-about/340_2017_perdue_biotechreg

^5^FDA (2020). Coronavirus Treatment Acceleration Program (CTAP). Accessed July 17, 2020. https://www.fda.gov/drugs/coronavirus-covid-19-drugs/coronavirus-treatment-acceleration-program-ctap

^6^MHRA regulatory flexibilities resulting from coronavirus (COVID-19). Accessed July 17, 2020. https://www.gov.uk/guidance/mhra-regulatory-flexibilities-resulting-from-coronavirus-covid-19

^7^Medicago. Accessed July 17, 2020, https://www.medicago.com/en/pipeline/; iBio Inc. Accessed July 17, 2020, https://www.ibioinc.com/therapeutics-and-vaccines; and Kentucky BioProcessing Inc. Accessed July 17, 2020; https://www.kentuckybioprocessing.com/

^8^WHO 2020. DRAFT landscape of COVID-19 candidate vaccines. Accessed July 20, 2020. https://www.who.int/publications/m/item/draft-landscape-of-covid-19-candidate-vaccines

^9^Press release March 12, 2020. https://www.medicago.com/en/newsroom/medicago-announces-production-of-a-viable-vaccine-candidate-for-covid-19/ Press release July 14, 2020. https://www.medicago.com/en/newsroom/medicago-begins-phase-i-clinical-trials-for-its-covid-19-vaccine-candidate/

^10^iBio Inc. Press release June 4, 2020. https://www.ibioinc.com/news/ibio-announces-second-covid-19-vaccine-program

^11^British American Tobacco. Press release April 1, 2020. https://www.bat.com/group/sites/UK__9D9KCY.nsf/vwPagesWebLive/DOBN8QNL

^12^https://www.nytimes.com/2020/08/01/world/asia/coronavirus-vaccine-india.html

^13^Fact Sheet: Explaining Operation Warp Speed. Accessed July 18, 2020. https://www.hhs.gov/about/news/2020/06/16/fact-sheet-explaining-operation-warp-speed.html

^14^https://www.nytimes.com/2020/07/31/health/covid-19-vaccine-sanofi-gsk.html

^15^Press release March 21, 2020. https://www.medicago.com/en/newsroom/government-of-quebec-providing-7-million-towards-medicagou2019s-covid-19-vaccine-development/ News release by Government of Canada March 23, 2020. https://pm.gc.ca/en/news/news-releases/2020/03/23/canadas-plan-mobilize-science-fight-covid-19

^16^Emergency Use Authorization. Accessed July 20, 2020. https://www.fda.gov/emergency-preparedness-and-response/mcm-legal-regulatory-and-policy-framework/emergency-use-authorization#covid19euas

^17^Animal Rule. Accessed July 20, 2020. https://www.fda.gov/drugs/nda-and-bla-approvals/animal-rule-approvals

^18^Mapp Biopharmaceutical website. Accessed July 15, 2020. https://mappbio.com/

^19^https://www.who.int/publications/m/item/draft-landscape-of-covid-19-candidate-vaccines

^20^https://www.forbes.com/sites/stevensalzberg/2020/08/02/start-vaccinating-now/#49550ee6cf6e

^21^https://investor.lilly.com/news-releases/news-release-details/lilly-initiates-phase-3-trial-ly-cov555-prevention-covid-19-long

^22^https://pubmed.ncbi.nlm.nih.gov/

^23^https://www.who.int/publications/10-year-review/chapter-medicines.pdf?ua=1

^24^https://healthpolicy.duke.edu/publications/covid-19-manufacturing-monoclonal-antibodies

^25^Press release April 1, 2020. https://www.bat.com/group/sites/UK__9D9KCY.nsf/vwPagesWebLive/DOBN8QNL

^26^https://www.bizjournals.com/sanfrancisco/news/2020/07/23/covid-coronavirus-doj-genentech-antibody-amgen.html

^27^Deutsche Welle, accessed July 21, 2020, https://www.dw.com/en/the-coronavirus-economy-switching-production-for-the-greater-good/a-52852712
